# Reflecting on the challenges encountered by nurses at the great Kermanshah earthquake: a qualitative study

**DOI:** 10.1186/s12912-021-00605-3

**Published:** 2021-06-07

**Authors:** Alireza Abdi, Aliakbar Vaisi -Raygani, Bahareh Najafi, Hamid Saidi, Khalil Moradi

**Affiliations:** 1 Emergency and Critical Care Department, Nursing and Midwifery School, Kermanshah University of Medical Sciences, Kermanshah, Iran; 2 School of Nursing and Midwifery, Kermanshah University of Medical Sciences, Kermanshah, Iran; 3 School of Nursing and Midwifery, Kurdistan University of Medical Sciences, Sanandaj, Iran; 4 School of Nursing and Midwifery, West Azerbaijan University of Medical Sciences, Urmia, Iran; 5 School of Nursing and Midwifery, Urmia University of Medical Sciences, Urmia, Iran

**Keywords:** Earthquake, Nurses, Experiences, Qualitative study, Iran

## Abstract

**Background:**

Iran has experienced an increasing number of earthquake in the past three decades. Nurses are the largest group of healthcare providers that play an important role in responding to disasters. Based on previous studies, they experienced challenges providing care in the previous disasters. Therefore, this study aimed to explore the nursing challenges to provide care to the injured in the Kermanshah earthquake, Iran.

**Methods:**

This is a qualitative study with conventional content analysis using Granheim and Landman approach. In this study, 16 nurses involved in providing care to the injured in the Kermanshah earthquake were selected by purposeful sampling method. Data were collected using in-depth semi-structured interviews. The criteria proposed by Guba and Lincoln were used to ensure the validity of the study.

**Results:**

Data analysis led to the emergence of 453 primary codes, 14 subcategories, and 5 categories. The five categories were as follows: (a) organizational and managerial challenges; (b) human resources; (c) infrastructure; (d) educational preparations; (e) and ethical.

**Conclusions:**

The results of this study showed that nurses faced several challenges in providing care to earthquake victims. Based on these findings, better educational management and planning, infrastructure reform, and establishment of a crisis nursing national team seem necessary.

**Supplementary Information:**

The online version contains supplementary material available at 10.1186/s12912-021-00605-3.

## Background

Earthquake is one of the most destructive natural disasters. Based on the international Emergency Events Database (EM-DAT), the rate of earthquakes is growing all around the world so that 792 cases have been reported between 1987 and 2015 [1]. As a developing country in Asia, Iran is one of the top 10 seismic countries in the world [2]. On the night of 12th of November 2017 at 21:48 local time, a devastating earthquake magnitude 7.3 on the Richter scale struck the region near the Iran–Iraq border in the West of Iran [3]. In this event, there were 620 deaths, 8,000 wounded, 70,000 homeless, 4,700,000 affected by it, and ≥ 12,000 damaged buildings [4]. After a natural disaster incidence, a surge in the demand for health and treatment services takes place [5] significance of nurses during a disaster is obvious since nurses are usually and constantly involved in disaster response by being the biggest group of human resources of health care providers in disasters [6]. Nurses often work in difficult conditions with restricted resources and always have more problems and challenges in their work [7]. Studies have shown that the nurses who provide care services to earthquake victims encounter several challenges like resource shortages [8], personal and family safety concerns [9], health problem management [10], and moral challenges [11]. Given the high risk of earthquakes in different regions of Iran and involving the nurses in these crises [9, 12], it is essential to have fundamental insights about these challenges. The issues and challenges faced by nurses in providing care to earthquake victims have not been examined comprehensively [13]. Based on the experiences of the participants, we can achieve a basis for programming and policymaking in health care systems to face critical situations. The present study elaborated on the challenges faced with by nurses in providing care to the victims of the Kermanshah earthquake 2017.

## Methods

### Design

A qualitative approach using conventional content analysis was performed for this study. The content analysis is an interpretation of meaning to recognize codes, sub-categories, categories, and themes [14].

### Sampling and study setting

We purposively selected nurses with an experience of providing care to the victims of the recent Kermanshah earthquake. In our study, the interviews were continued to reach the saturation point, which was obtained by interviewing 14 nurses, but two additional interviews were performed to make certain that no new concepts were developed, so there were 16 interviews in total. To achieve the maximum variability, the participants were recruited from several teaching hospitals affiliated with Kermanshah University of Medical Science (Especially Shohada Hospital in Sarpol-e Zahab, Imam Reza Hospital and Talegani Hospital) and other provinces (Tabriz, Tehran, Isfahan, Kurdistan, and Shiraz in Iran). These cities were selected due to they had the most presence and cooperation. Researcher, to select qualified participants and access information necessary was present in these places.

#### Characteristics of participants considered for inclusion

(1) Having a direct role in providing care to the earthquake victims for at least 24 h in total in the region hit by the earthquake. (2) Having at least 1 year of work experience in clinical nursing practice. (3) Willingness to participate in the study.

### Participants

Mean age of the participants was 34.12 ± 5.77 years, with mean work experience of 10.75 ± 5.3 years. The majority of them were married, nine participants (56.25 %) were women and the rest were men. Eleven participants (68.75 %) had a bachelor’s degree and the rest had a master’s degree and other than the Kermanshah earthquake had no previous experience in disaster relief work. A total of 16 participants came from emergency, internal, surgical, cardiac care unit, and intensive care units, and all had more than 3 years of work experience. Eight participants entered the disaster site within 36 h, they voluntarily joined rescue teams and were deployed to the most affected areas, which were Sarpol-e Zahab City and Salas-e Babajani City. The mean time period spent in the earthquake hit region was 9.18 + 3.65 days (see Table 1).

**Table 1:** Descriptive characteristic of the participants

Participant No.	Education Level	Ward	Working Experience (Years)	Lengths of stay(days)
N1	BSc	Emergency	7	8
N2	MSc	Internal	16	5
N3	BSc	Emergency	9	7
N4	BSc	ICU	5	11
N5	BSc	Surgical	12	5
N6	MSc	Emergency	9	13
N7	BSc	Surgical	12	14
N8	MSc	CCU	18	3
N9	BSc	Internal	11	7
N10	BSc	ICU	16	14
N11	BSc	Emergency	4	9
N12	BSc	Surgical	13	10
N13	BSc	offices	22	15
N14	MSc	ICU	8	7
N15	BSc	Emergency	3	7
N16	MSc	Emergency	7	12

### Data collection

Face-to-face, semi-structured, in-depth interviews and written field notes were conducted by the corresponding author who was a PhD candidate in Nursing. Each interview took from 30 to 82 min (61 minutes on average) and was conducted at the nurses’ workplaces in rooms where it was possible to talk privately. The interview started with questions regarding demographic data and was followed by a general question such as “What challenges did you face when providing care to the injured?” To gain clearer and in-depth information, probing questions such as “Can you give an example of this? Or " Can you explain more about this?” were asked. All interviews were recorded digitally with the permission of the participants, transcribed verbatim, and imported into MAXQDA software (version10) for data management. The participants were interviewed by the face-to-face method in Persian language, all interviews by a translator fluent in English and Persian translated into English. Each interview was reviewed and analyzed by two authors, and the retrieved information used a guide for the next interview with a new participant.Two participants refused to continue the interview due to recalling unpleasant memories while providing care earthquake. With the permission of these two participants, they were referred to a consultant.

### Data analysis

Data analysis was conducted simultaneously with the interviews based on the methods proposed by Lundman and Graneheim [15]. After each session, the recorded contents of interviews were repeatedly and carefully listened to, transcribed on paper, and then typed in Microsoft Word, and data were classified and analyzed in MAXQDA software10.0R250412. The semantic units were identified and encoded after careful review of the transcribed materials.The codes were then reviewed several times in a continuous process from code extraction to labeling. Similar codes were merged, categorized, and labeled and the subcategories were determined. The extracted subcategories were finally compared and merged to form the main categories.The researcher’s (Corresponding Author: khalil moradi) had 9 years of work experience in the emergency department. This helped them to identify implicit meanings in the participants’ responses.To avert insider bias and make sure not to direct or effect the results of the study, the author used reflexivity by recording her experiences and feelings in a diary.

### Trustworthiness

To assure study trustworthiness, we considered four criteria throughout the study process: credibility, dependability, conformability, and transferability [16]. To increase credibility, the researchers performed member checks with participants during the process of data collection and analysis. For member checking technique, the participants reviewed the content of the interview, codes and themes extracted from the interview to ensure the accurate meaning and for really reflecting their experiences. In addition, to support the credibility employing the peer checking techniques, long-term and ongoing engagement with the data were used. For dependability, the raw data, codes and subcategories were saved for audit purposes, and all procedures of the study and details were noted and recorded. To establish conformability, researchers shared reflective manuscripts on the research subject, permitting researchers to acknowledge prior experiences and understandings of the phenomena. In addition, researchers conscientiously performed reflective thinking to bracket individual opinions and ways of thinking. The sampling with maximum diversity was used to enhance the transferability of findings.

## Results

Data analysis led to the development of 5 categories and 14 subcategories. The categories emerging from the data analysis were included (1) Organizational and managerial challenges (2) human resources (3) infrastructure (4) educational system, and (5) ethical. These categories with their subcategories are explained in the following tables (Tables 1 and 2).

**Table 2:** Theme, categories, and sub-categories extracted from data

Theme	Category	Subcategory
**Nursing care challenges in earthquake**	**Organizational and managerial**	1. Insufficient coordination among health team members 2. Lack of unity in command 3. Inadequate Organizational Management
	**Human resources**	1.Weakness in provision of occupational health for the nurses 2. Poor management of volunteers 3. Lack of uniforms for health workers 4. Nurses’ concern for their own families
	**Infrastructure**	1. Communication disruption 2. Vulnerability of local health facilities 3. Difficult access
	**Educational preparations**	1. Nurses’ poor knowledge in the field of disaster 2. Lack of comprehensive training program
	**Ethical**	1. Ethical challenges related to prioritizing injured 2.Ethical challenges due to lack of resources

### Organizational and managerial challenges

This category represents the participants’ statements about the absence of a concentrated management and programming system and poor coordination among the organizations that provided services during disasters, which lead to the following subcategories:

#### Insufficient coordination among health team members

The field hospitals available in the region were not affiliated with one specific organization. They were established by different organizations and had different equipment with no coordination and arrangement among them. Because of this, the financial and human resources were not used efficiently and, in many cases, continuity of services was halted.

One participant clearly said that*“The field hospitals established by the army and the University of Medical Sciences were at different places. The army hospital was fully equipped and located away from the Sar Pol Zahab mobile hospital. However, nobody knew that it was there and for a simple chest-x-ray we had to dispatch patients to Kermanshah by a helicopter” (P10).*

Another participant mentioned: *“There were several medical teams in some places and rural area in particular, while there were none in other places” (P5).*

#### Lack of unity in command

The nurses who experienced the Kermanshah earthquake mentioned issues and challenges like inconsistency between the requested medicines and supplied items, several command authorities, and intervention by policy makers and state authorities. This indicates a lack of unity in command, which was an issue in providing services to the victims.

This situation confuses most nurses and often, parallelism and disorder is due to the large number of managers and commanders in the environment, whose existence had made it difficult to provide appropriate services.

For example, a participant stated that *“In many cases, serums and medicines would be supplied by other provinces without supervision and need assessment so that the large supply of unrequired medicine would only limit our operation spaces” (P13).*

Another participant expressed: *“There were several authorities who had different strategies” (P1).*

#### Inadequate organizational management

The nurses highlighted chaos and overlapping of operations, ambiguity of tasks, and conflicts of interest among organizations due to the obscurity of roles. This indicates negligence of the importance of organizing.

A participant stated that *“The Red Crescent is not directly the medical team, but they had erected their tents inside the hospital and converted the space into a place for distributing baby formula, clothes… and it was not easy for us to tell if someone needed medical attention or not. They also intervened with nursing services and created more problem for the nursing personnel” (P13).*

### Human resources

Challenges related human resources was another main category that was highlighted in many interviews. Challenges related human resources includes four sub-categories: weakness in provision of occupational health for the nurses, poor management of volunteers, lack of uniforms for health workers, and nurses’ concern for their own families.

#### Weakness in provision of occupational health for the nurses

Negligence of physical and mental health of the nurses was an issue. The majority of nurses would work nonstop; however, there were no proper welfare facilities for them. This lowered productivity of the workforces. In this regard, the participants stated:

*“Because of the severity of the damages to the region, our nurses had almost lost their spirit. However, mental health of nurses was not important for anyone. The nurses were lost themselves” (P3).*

*“We did not have lavatory during the first 48 hours. There was no rotating work system or facilities for nurses, we had no place to sleep” (P15)*.

#### Poor management of volunteers

Lack of attention to the organization of dispatch teams and their proper use, Uncertainty of their capabilities and improper deployment and distribution of relief forces in the affected areas, leads to inefficient performance of personnel. This finding was highlighted in the following statement:

“*There were many nurses from different provinces who were not put in use properly. The least they could do was to let the local nurses to use vacation for a week to handle their personal affairs” (P7).*

Based on the experiences of the participants, Forces that do not have the necessary competence and readiness to conduct care in a crisis situation should not be used.

Another participant expressed: *“Nursing students did not have any specific skills, they would gather around a bed trying to find a vein, but all they would do was causing more harm to the patient” (P2).*

#### Lack of uniforms for health workers

Based on the experiences of the participants, Lack of identification and distinction between individuals as well as professional and specialized teams due to lack of identification card or appropriate uniforms leads to abuse in various forms (for example, Theft of drugs by people who introduced themselves as doctors) and not recognizing service providers in earthquake-stricken areas for people and authorities.

One of the nurses said: “*Many nurses and physicians in the region did not have an ID card or a uniform. There was this guy who claimed to be a pediatrician and took medicines to villages nearby. Later we found that he was a welder” (P4).*

#### Nurses’ concern for their own families

The first concern for the local nurses was their families and their safety. This issue had somehow engaged their minds and led to more confusion for nurses and lack of focus in providing services. In this regard, the participants stated:

*“First, you need to make sure about your own family; otherwise, the concern affects your work. How can I stay at work when I am not sure if my family is alive or not” (P4).*

Another participant mentioned: *“My baby and husband were in the car outside the hospital. Every few hours I would return to them to breastfed my baby and then return to work. I was highly under pressure” (p8).*

### Infrastructure

Another one of challenge of nursing care in earthquakes was the challenges related to infrastructure. This category includes three sub-categories: communication disruption, vulnerability of local health facilities, and difficult access. The findings showed that the participants encountered several challenges in this regard.

#### Communication disruption

Immediately after earthquake, the telecommunication services in the region were halted so that communication for making arrangements to manage the crisis was a serious challenge for the personnel.

*A participant said that “The telephone service was off and mobiles were not working. It was very hard to contact the province crisis control center and other relief centers” (P1).*

#### Vulnerability of local health facilities

Despite serious damages to the health infrastructure, Chaos in primary medial aids, power outage, and darkness, nurses provided health care in such unfavorable conditions.

One of the participants noted: “*After the earthquake, almost all health care centers were out of commission. The staff would provide health service at the hospital yard (very cold weather) using their mobile light” (P14).*

#### Difficult access

Heavy road traffic due to the stampede and massive destruction on the streets near the hospital in particular was one of the main causes of disorder in providing health care by nurses. In this regard, one of them stated that “It was *a real mess. There were many injured on the way to the hospital who were stuck in the traffic 1km away from the hospital. We would have been more helpful if we had access to the injured” (P12).*

### Educational preparations

Another one of challenge of nursing care in earthquakes was the challenges related to education preparations. Interviews showed that nurses were not prepared to face disasters and had not received adequate education in this area, which lead to the following subcategories: nurses’ poor knowledge in the field of disaster, and lack of comprehensive training program.

#### Nurses’ poor knowledge in the field of disaster

Many of the participants acknowledged their lack of knowledge and skill for providing care to earthquake victims. They highlight this as a critical care void in their profession. In this regard, the participants stated:

“*The nurses were not familiar with the protocols of carrying patient, safety, and flight physiology. They did not know how to carry patients by helicopter” (P8).*

Another participant expressed: *“Many of the injured had suffered crush syndrome; however, most of the nurses knew nothing about it” (P9).*

#### Lack of comprehensive training program

The nurses mentioned their lack of readiness and they believed that it was due to a gap in the formal education system and in-service educations.

One participant highlighted this: “*We had no education about crisis; all we had was a two-credit course in the bachelors’ program” (P11).*

Another nurse expressed: *“Due to the lack of integrity and harmony in educational programs on crises, our nurses were not able to demonstrate their true capabilities during the crisis” (P6).*

### Ethical

 Almost all the participants mentioned moral challenges they faced when providing health cares to the earthquake victims. They had also found these challenges disturbing optimal care. For this reason, their statements revealed following subcategories.

#### Ethical challenges related to prioritizing injured

As revealed by the interviews, when the disaster is large in scale, the nurses face hard decision-making situations. In many cases, they have to make unfair and unpleasant decisions and in some cases these decisions are about life or death. In this regard, the participants stated:

“*It was a very frustrating situation, it was not easy to decide which one should be attended first, that child, that adult or that elderly….” (P5).*

One of the participants noted: *“One of the victims had an apnea and we needed resuscitation trolly, aftershocks would not stop and the building was collapsing. It was not able to decide whether or not should I risk my life and go inside the building to fetch the trolley” (P11).*

#### Ethical challenges due to lack of resources

During disasters, material and human resources scarcity is common. As a result, staff being unable to provide all services in the event of a disaster, this creates specific moral dilemmas for the nurses. The nurses talked about their experiences as conflicts between their knowledge of standard performance and failure to meet the standard. This was a source of moral challenge for them.

One of the nurses stated: “*We had to use one forceps for several patients with cuts; I really do not know if our work was ethical or not?” (p14).*

## Discussion

In this study, the nurses’ experience regarding the challenges faced of providing care to earthquake victims was examined. Semi-structured interviews were performed and the data analysis yielded five categories. Organizational and managerial challenge was one of the main extracted categories. According to the participants, different organizations like the ministry of health, the Red Crescent Society, and other relief organizations were in responsible dispatching health, treatment and service forces to offer services to the disaster areas.These organizations worked independently and there was no coordination among them and each of them considered themselves as the leader of providing services in this conditions.

The finding of this study was supported by previous studies. In Pouraghaei study conducted Azerbaijan earthquake in 2012, it was revealed that the lack of coordination among organizations as one of the main challenges [17]. Another study by Li et al. on Sichuan earthquake in China, pointed to the lack of collaboration among disaster relief teams and insufficient leadership [18]. Apparently, one of the challenges of modern age, given the type of disasters, is the necessity of an integrated system to face disasters.

Human resources challenge was another extracted category in this study. The participants highlighted issues like lack of welfare facilities for care givers like accommodation facilities, nutrition, bathrooms, and negligence of mental health of nurses. Other studies have reported similar concerns in nurses like personal safety, adequate food, water, and accommodations [19, 20]. Hugelius et al. noted that providing psychological support was one of the main elements in nursing services in the face of disasters [21]. In addition, Salmani et al. noted that volunteers’ potential problems and their physical and mental status should be assessed during and after disasters [22]. The participant mentioned ineffective call and distribution of relief forces in the regions hit by the earthquake, lack of uniform, and their concerns about safety of their families. Studies on Sichuan earthquake in China and the Bam earthquake have highlighted poor human resource organization as well [18, 19]. Despite the fact that health and treatment personnel were present in the region, they were not known to officials and clients in the early days. In the case of the Bam and Azerbaijan earthquakes, some of the volunteers did not have uniform and ID card [17, 19]. When providing care in critical situations, providing dress is fundamental to distinguish the health providers.

Despite the valuable role of nurses in the earthquake region, they and their families received no mental, financial, and emotional support. Similar problems have been reported in other studies [9, 17, 23]. Nursing Organization can take steps in this regard by providing protection and support for nurses and their families during disasters as incentives for nurses. Such services solve the concerns in nurses and improve their capability to provide health services.

Another main category was infrastructural challenges. The participants had experienced several challenges in this field. Ardalan et al. reported that due to communicational problems and the demolition of three health centers and 89 local clinics failed to play an efficient role during the first phase of reaction to Azerbaijan earthquake [24]. Infrastructure disruption after disasters have been mentioned in several study as a serious challenge [25, 26].

Another extracted challenge was about education system. Many of the participants acknowledged the lack of skills and knowledge for providing health care in disaster and absence of a comprehensive education program in this regard. A systematic review in 2017 showed that nursing curricula has a limited amount of disaster-related content, so they were not ready to work in disasters; most of the reviewed studies were from Asian countries [27].

 Like many other countries, crisis nursing education is not completely developed in Iran and nurses do not have the knowledge and skill to work in disasters due to lack of education [13]. Empowerment of nurses and improvement of their awareness can be a major step to provide timely and proper services to victims during disasters. Theoretical and practical educations should be a part of nursing program to improve performance of nurses during disaster.

Another category based on the experiences of nurses was moral challenges. Some of the participants mentioned situations full of moral challenges and that they had no education in this regard. Moral challenges in providing care to the victims while the safety of nurse is at risk were discussed. These findings are consistent with other studies on nurses who helped earthquake victims and faced with moral challenges [9, 11, 28]. Studies have shown that serious lack of resources and hard situations create moral challenges like whether or not washing equipment with drinking water is a right thing to do. Similarly, nurses in our study also reported such practices as using one forceps for multiple injuries [8, 29]. Moral preparedness of nurses who are the front line of providing care services during disaster neglected and they suffer the consequences of different professional and moral challenges afterwards [9].

## Limitations

There are several limitations that should be considered when interpreting the findings of study. The main limitation of qualitative studies is the lack of generalizability of the results, to which our study is not an exception. The results may be specific to this environment and culture.The study findings are a reflection of the experiences of a small group of nurses attending an earthquake zone in one province of Iran. To alleviate this limitation, the participants were selected from different provinces with maximum diversity. Despite providing rich information of the concept under study, the time gap between the actual experiences and the interviews could also cause concerns about accuracy of the recollections.

## Conclusion

The nurses faced with several challenges in providing care to earthquake victims. Five main categories of these challenges were extracted that need serious, efficient, and effective intervention to ensure better care services in similar situations in the future. Similar challenges had been experienced in previous earthquakes like the Roudbar, Bam, and Azerbaijan earthquakes. But few lessons from these earthquakes have been consistently applied in earthquake disaster management practices. Many of the lessons from the great earthquake disasters of previous did not become ‘‘Lessons Learned. ’’ To the contrary, these lessons were identified again in Kermanshah earthquake in Iran. In other words, we re-experience the experience. Given the crisis nursing status in Iran, better curriculum planning and implementation, revision of nursing curriculum, and establishing crisis nursing national team (professional pre-identified volunteers) are essential measures to improve professional competence of nurses in the face of disasters.

## Supplementary information

**Additional file 1.** Interview Guide.

## Data Availability

Data are available by contacting the corresponding author.

## Abbreviations

BSc: Bachelor of Science
MSc: Master of Science
ICU: Intensive care unit
CCU: Cardiac care unit
