# Supplementary material for: Reflecting on the challenges encountered by nurses at the great Kermanshah earthquake: a qualitative study
Source: BMC Nurs. 2021 Jun 7;20:90. doi: 10.1186/s12912-021-00605-3 (PMC8185949; doi:10.1186/s12912-021-00605-3)
Supplement: Supplementary file 1 — Additional file 1. Interview Guide. [file 12912_2021_605_MOESM1_ESM.docx]

**Additional file: Interview Guide**

| **Box 1. Semi-structured interview guide**  In this qualitative study, semi-structured individual interviews were used to collect data. In this type of interview, the questions varied according to the participants' answers. However, here are some of the questions that guided the interview in the present study and below these questions, It is mentioned as follows:  "Please explain your experiences about providing care to the injuries of the Kermanshah earthquake."  What challenges did you face when providing care to the injured?  Can you give an example of this? Or "Can you explain more about this?  Is there anything else you would like to explain? |
| --- |
